# Effectiveness of the attitudes and knowledge of under graduation students after the application of an online educational module: Environmental protection in dentistry

**DOI:** 10.21142/2523-2754-1204-2024-215

**Published:** 2024-11-23

**Authors:** Carmen Rosa García Rupaya, Nimia Olimpia Peltroche Adrianzén, Johan Caballero García

**Affiliations:** 1 Facultad de Odontologia, Universidad Nacional Federico Villarreal. Lima, Peru. cgarcia@unfv.edu.pe, npeltroche@unfv.edu.pe Universidad Nacional Federico Villarreal Facultad de Odontologia Universidad Nacional Federico Villarreal Lima Peru cgarcia@unfv.edu.pe npeltroche@unfv.edu.pe; 2 Facultad de Ciencias de la Comunicacion, Universidad Tecnologica del Peru. Lima, Peru. jcgfotografia@outlook.es Universidad Tecnológica del Perú Facultad de Ciencias de la Comunicacion Universidad Tecnologica del Peru Lima Peru jcgfotografia@outlook.es

**Keywords:** environment, dentistry, video, education, medio ambiente, odontología, vídeo, educación

## Abstract

**Materials and Method:**

the design was prospective, longitudinal, analytical, and quasi-experimental. The sample was census type, consisting of 99 students from the third semester, 61 students from the fifth semester, and 48 students from the ninth semester. An educational video on environmental care was prepared with the participation of representatives of the Ministry of the Environment, authorities of the faculty and the university of social responsibility. Likewise, a questionnaire was developed and validated to measure attitudes and knowledge about caring for the environment in the dental field. Sociodemographic variables will also be considered. The instrument was applied before and after the educational program.

**Results:**

in the pre-application of the educational program, attitudes had an average score of 17.30 and knowledge had an average score of 4.33. When comparing the scores, in the pre-application, between men and women for attitudes, they were statistically significant (p=0.0017), as for the semester, in attitudes (p=0.021) and knowledge (p=0.0102), significant differences were found. When comparing the attitude scores in the post-application increased, and no differences were observed according to gender or semester. However, significant differences in knowledge were observed depending on the semester (p=0.0001).

**Conclusion:**

The Online Educational Program *‘Environmental Care in the Dental field’* was effective in improving attitudes and knowledge according to pre- and post-application comparisons.

## RESUMEN

**Objetivo:**

Identificar la efectividad en el conocimiento y las actitudes de estudiantes de odontología tras la aplicación de un módulo educativo en línea: Cuidado del Medio Ambiente en el Ámbito Odontológico.

**Metodología:**

El diseño fue prospectivo, longitudinal, analítico, cuasi experimental. La muestra fue de tipo censal y estuvo conformada por 99 alumnos del tercer semestre, 61 alumnos del quinto semestre y 48 alumnos del noveno semestre. Se elaboró un video educativo sobre el cuidado del medio ambiente, con la participación de representantes del Ministerio del Ambiente, autoridades de la facultad y de la universidad de responsabilidad social. Asimismo, se elaboró y validó un cuestionario para medir las actitudes y el conocimiento acerca del cuidado del medio ambiente en el ámbito odontológico. Se consideraron, además, variables sociodemográficas. El instrumento se aplicó antes y después del programa educativo.

**Resultados:**

En la preaplicación del programa educativo, las actitudes tuvieron una puntuación media de 17,30 y los conocimientos, una media de 4,33. Al comparar los puntajes, en la preaplicación, entre hombres y mujeres para las actitudes fueron estadísticamente significativos (p = 0,0017), en cuanto al semestre, en actitudes (p = 0,021) y conocimientos (p = 0,0102) se encontraron diferencias significativas. Al comparar los puntajes de actitudes posaplicación, estos se incrementaron y no se observaron diferencias según sexo ni semestre. Sin embargo, sí se hallaron diferencias significativas en conocimientos según el semestre (0,0001).

**Conclusión:**

El Programa Educativo en Línea: Cuidado del Medio Ambiente en el ámbito odontológico, fue efectivo para mejorar las actitudes y los conocimientos, de acuerdo con las comparaciones pre y posaplicación.

## INTRODUCTION

There is a notable growth in social responsibility and care for the environment. This task cuts across all professions and must generate a paradigm shift in the daily actions of each person. Several publications reported that environmental problems accumulate and grow over time, resulting from little or no knowledge and indifferent and inadequate behaviors, behaviors, and attitudes toward these problems.^1^^,^^2^

The prestigious journal ‘The Lancet’ published that, among other aspects, climate change greatly affects health, which must be considered for the best care of the environment.^3^ climate change triggers extreme temperatures in our environment, and water and air pollution also affects the population's health.^4^ If we do not become aware of these phenomena that occur in the world, the crisis for people's health will increase, hence the importance of taking appropriate measures for control with social and environmental adaptation.^5^

 Recently, there has been a lot of concern about this issue; government institutions in the country, such as the Ministry of the Environment (MINAM), handle a lot of information of this nature through official documents and dissemination through social networks. However, the public, to which one is addressed, does not show much interest in the matter.^6^

Herrera et al.^7^ refer to lifestyles in a “sustainable” way aimed at developing pro-ecological behavior by supporting fundamental aspects such as saving water and electricity, recycling, and avoiding procedures that contaminate the environment so that it is oriented to environmental care.

 On the other hand, Kaiser et al.^8^ define “pro-environmental behavior as actions that contribute to the preservation and conservation of the environment.”

In a study carried out in Peru, the type of relationship between behavior and responsible environmental attitude was identified in a sample of young people from a university in Lima. These researchers maintained that attitude towards the environment could be an indicator of behavior and can produce actions towards the application of ‘pro-environmental regulations.^9^

In waste management, priority must be given to the protection of health personnel and the population in such a way as to reduce the impact that may arise due to environmental exposure. Health professionals must raise awareness to prevent the spread of diseases based on contaminating waste.^10^ Nowadays, social networks reach many people, and technological means such as online communication are a very versatile because more people can participate without limits on location or time. In turn, this tool can promote a change of behavior through the dissemination of knowledge.^11^

A study was conducted in the United Kingdom to design and evaluate an online module for third- and fourth-year UK medical students entitled *'Climate change and sustainability in clinical practice.'* Among the secondary objectives, students' attitudes toward climate change and sustainability in healthcare education were evaluated. This background supports the before and after the procedure of applying an online educational module on climate change and how this improves the knowledge and attitudes of future doctors.^12^

The health sector provides care in various hospitals, clinics, and medical and dental faculties that produce a large amount of biomedical waste, including human anatomical waste, material contaminated with blood and oral fluids, syringes, needles, etc., triggering significant environmental contamination.^13^ Unfortunately, not many people reflect on this issue. In the case of dental students who learn throughout their training the management of all types of contaminating waste as well as are exposed to radiation and toxic materials, they must know the best care for their health and the health for their patients.

Since dentistry is considered a health science and given that it has a direct contact with patients and the community in general, it is necessary to reflect on caring for the environment from our professional perspective. Educational programs are carried out with the aim of improving behaviors and attitudes towards self-care in health, to the extent that these interventions are carried out digitally, they can have a greater reach.^14^ Selecting a better way to access studies using the information technology, and an online educational program properly designed with valuable information but that at the same time reaches this target audience in an understandable and practical way; and above all in a more dynamic and versatile way since there are no time or location limits.

Therefore, the aim of this research was to identify the pro-environmental knowledge and attitudes that dental students have after the application of the Online Educational Module: Environmental care in the dental field.

## MATERIALS AND METHOD

The research was carried out at the headquarters of the Dental School of the Federico Villarreal University (FO-UNFV), during the 2023 academic year, third, fifth and ninth semester students of the FO-UNFV participated. The project received approval from ethics committee number 006-2023-CE-FPSI-UNFV. The sample was made up of 208 students. For the present study, an instrument was developed based on published scientific literature, among which is the Ministry of the Environment (MINAM), as well as a questionnaire validated in the research “Environmental knowledge and pro-environmental behavior of high school students, in the Valparaíso Region” by the authors Barazarte Castro et al.^15^, which includes attitudes and knowledge towards pro-environmental behavior. To validate the questionnaire, the judgment of experts was carried out who evaluated the content, order, clarity, sequence and time of the developed questionnaire. Then they filled out the respective evaluation form, obtaining an Aiken V with a score of 0.95.

Regarding the data collection techniques in the first and second stages, it was face-to-face. Data corresponding to the covariates (gender, age, semester of study and place of residence) were also included. The before and after methodology was applied to the application of the online educational program in relation to caring for the environment, this was based on the study carried out by Dunne et al.^16^ in 2022 at the University of Cambridge in the United Kingdom, who also developed an online module in relation to climate change aimed at medical students with the aim of improving their knowledge and attitudes on this topic.

For the preparation of the Online Educational Module: Care of the Environment in the Dental field, interviews were carried out with authorities in the area of social responsibility of the Federico Villarreal University and representatives of the Directorate of Education and Environmental Citizenship of the Ministry of the Environment. The educational program (video media) that was developed for this research contains the following information: Section 1- Protection of the environment in the country and at UNFV Section 2 - Care of the environment in Dentistry, which includes topics on the management of contaminating waste, management of single-use plastics, energy saving measures, reduction of paper use, reduction of carbon dioxide emissions and biosafety in the dental clinic. The educational module is original and has been prepared especially for this research. The team included the participation of an audiovisual communicator who made the recordings, music, colorization and editing of the content of the material.

Before taking the surveys, an Informed Consent Sheet was filled in person, subsequently the questionnaire was also filled in person, all of these activities were prior to participation in the educational program. Subsequently, once the educational module was prepared, it was projected in real time to the students at times coordinated with the teachers and students. Subsequently, the instrument was applied again to the students; It is worth mentioning that two open questions were added in relation to caring for the environment in their daily life and in the dental field. The questionnaires were anonymous and have been treated confidentially. The project was evaluated by the Ethics Committee of the Health Sciences Research Unit.

### Statistical analysis

The answers provided in the initial and final questionnaires were stored in an Excel database and processed with the STATA 18 Program. The attitude scores range from 5 to 25 points, and there are 5 statements (five points per item is the maximum) according to the Likert scale), while in knowledge (ten questions) the highest score is ten, with a maximum point per item. Absolute and relative frequencies of the qualitative study variables were obtained and means, and standard deviations were obtained for the quantitative variables. To compare the scores obtained in attitudes and knowledge in the pre-application and post-application stages according to gender and semester of study, the Mann Whitney U and Kruskall-Wallis tests were applied. Spearman's correlation was applied to relate the pre- and post-application knowledge and attitude scores of the educational program. A significance level of α ≤ 0.05 was used.

## RESULTS

In Table 1 you can see the characteristics of the sample, the largest group 47.60% corresponds to students in the third semester, mostly women (67.79%) and the mean age is 21 years. In Table 2, regarding the scores obtained, attitudes have an average score of 17.30 and knowledge an average of 4.33. In Table 3, it can be seen that the average highest score in attitudes and knowledge was obtained by female students, and also these results in the case of attitudes were statistically significant. Depending on the semester, it is observed that for knowledge the average of the highest score was obtained by those in the fifth semester with 4.70, and in attitudes with a score of 17.80. Both results were statistically significant. In Table 4, regarding the scores obtained, attitudes have an average score of 19.73 and knowledge an average of 7.88.

**Table 1:** Characteristics of the study sample

Variables	Absolute frequency		Relative frequency %	
Semester			
Third		99		47.60
Fifth		61		29.33
Ninth		48		23.08
Gender				
Males		67		32.21
Females		141		67.79
Age				
	Mean	21.25	SD	2.86

**Table 2:** Descriptions of the scores of knowledge and attitudes before the implementation of the educational program

Variables	Mean	SD	Minimum	Maximum
Score Attitudes	17.30	2.98	7	23
Score Knowledge	4.33	1.43	2	8

**Table 3:** Description and comparison of knowledge and attitudes according to gender and semester before the application of the educational program

Variables	Attitude Mean (SD)	p	Knowledge Mean (SD)	p
Gender			
Male	16.33 (3.21)		4.13 (1.37)	
Female	17.76 (2.76)	0.0017*	4.43 (1.45)	0.1617*
Semester				
Third	17.54 (2.75)		4.03 (1.29)	
Fifth	17.80 (2.89)		4.70 (1.55)	
Ninth	16.17 (3.33)	0.021^	4.48 (1.43)	0.0102^

* Mann Whitney U test

^ Kruskall Wallis test

**Table 4:** Description of scores of attitudes and knowledge after the implementation of the educational program

Variables	Mean	SD	Minimum	Maximum
ScoreAttitudes	19.73	2.23	15	24
ScoreKnowledge	7.88	1.18	6	10

Table 5 reflects those men and woman had high scores in attitudes and knowledge, not representing a statistical difference. Regarding the semester of study, the attitudes were independent, however in knowledge, significant differences were observed, with the students of the fifth semester obtaining the highest score. Table 6 reflects a positive correlation between attitudes and knowledge in the pre-application and post-application of the educational program, this being in both cases statistically significant with a p<0.05. Table 7 reflects significant differences between the pre and post application of the educational program in attitudes and knowledge.

**Table 5:** Description and comparison of knowledge and attitudes after the implementation of the educational program by gender and semester

Variables	Attitudes Mean (SD)	p	Knowledge Mean (SD)	p
Gender			
Male	20.05 (2.28)		7.880 (1.31)	
Female	19.58 (2.19)	0.204*	7.879 (1.12)	0.8830*
Semester				
Third	19.868 (2.37)		7.838 (1.20)	
Fifth	19.672 (2.13)		8.459 (0.98)	
Ninth	19.521 (2.07)	0.757^	7.229 (1.06)	0.0001^

* Mann Whitney U test

^ Kruskall Wallis test

**Table 6:** Relation of scores from attitudes and knowledge before the implementation of the educational program

Time	Observations	Rho	p
Pre-test	208	0.1673	0.0158
Attitudes and knowledge			
Number of observations			
Spearman’s correlation			
Post-test	208	0.2521	0.0002
Attitudes and knowledge			
Number of observations			
Spearman’s correlation			

**Table 7:** Comparison of the scores from attitudes and knowledge before and after the implementation of an educational program

Variables	Mean	SD	p
Attitudes		
Pre-test	17.30	2.98	
Post-test	19.73	2.23	<0.001
Knowledge			
Pre-test	4.33	1.43	
Post-test	7.88	1.18	<0.001

Mann Whitney’s U test

## DISCUSSION

It is suggested to reinforce knowledge about environmental consciousness, since it is a constant concern in various disciplines due to climate change that is bringing negative consequences on various economic, social, labor and health variables. Careers related to health show a lot of interest because from an epidemiological point of view, new diseases have appeared as a consequence of climate change. Researchers from the University of Cambridge maintain that the climate crisis can affect the physical and mental health of all communities in the world, but also, the provision of medical care includes materials that unfortunately generate greenhouse gas emissions.^16^

In fact, there are some authors who have referred to this with the proposal of establishing norms towards caring for the environment in the community from a social point of view. ^17^ Likewise, Japanese academics relate health to the planet through an analysis of the environmental awareness and behavior, concluding that health could become a predictive factor for developing pro-environmental attitudes.^18^

During university education it is necessary to promote care for the environment. This is a primary issue of various authors who support incorporating related information in the curricula of health careers.^19^^-^^22^ According to the existing concern between the relationship between health and environmental care; a video was prepared for this research project that included general topics on environmental care and topics related to the dental area, with the objective of measuring, in the pre-application and post-application of the educational module, the scores of attitudes and knowledge in dental students.

The instrument consisted of an attitude questionnaire that addressed aspects related to caring for the environment in daily life, and knowledge addressed aspects that included the dental field and the social environment. It was observed that prior to the implementation of the program, the knowledge scores were at a medium to low level; and after the implementation of the training program the scores increased, this agrees with a similar investigation where an educational module on environmental care was also applied carried out in Cambrigde where the researchers conclude that they observed a significant increase in knowledge.^12^ On the other hand, the present study correspond with a research in which educational interventions on environmentally sustainable habits were carried out and these promoted changes in behavior and knowledge in students in North Carolina in the United States^23^, with the results after the program being statistically significant in a similar way to what observed in this research.

Regarding attitudes, medium to high positive attitudes were found prior to the application of the program and after the educational program they rose to a higher score for our study, from which it can be deduced that the students' attitudes were reinforced after some information was provided. These results agree with those found on dental hygienists in the United States, where they found high values after an educational intervention and, in the same way that in this study, the results were statistically significant. ^23^ Instead, in the research carried out among university students in Chile on perceptions and behaviors towards the environment, it was found that there is an attitude of concern and also a good level of knowledge of environmental issues, however only 23% of young university students had demonstrated pro-environmental behaviors ^24^, unlike this study, we found high knowledge and attitudes also with high scores after the application of the program.

In the present investigation, the correlation between attitudes and knowledge pre- and post-application of the educational program was evaluated. In both cases it was statistically significant (p<0.05), however, the correlation values were weak; unlike a study carried out in Peru where attitude values were correlated with pro-environmental behavior where they also found statistical significance with high correlation values^9^; this may be because the knowledge may be more difficult for students to acquire. On the other hand, in research carried out in Tokyo they found that the intention to participate in pro-environmental behavior was negatively correlated with pro-environmental knowledge^18^; which differs from the present study since we found a weak but positive correlation between knowledge and attitudes.

Based on the results of this study, it is observed that dental students, the majority are committed to caring for the environment in their daily lives and in the dental environment, however, it is necessary to deepen education and institutional support to take concrete pro-environmental actions in dentistry; Similar results are presented in a research carried out in New Haven in the United States on health professionals, where they conclude that the impact on education will be obtained from greater research and contribution from the industry to reduce the carbon footprint caused by the materials that are used in health, modifying the consumption patterns of certain products through institutional regulations and policies.^25^

It should be highlighted that dental students form an important social group with decision-making capacity in the future can promote greater knowledge and guide positive attitudes towards caring for the environment in the future. Hence the importance of this research, which has chosen to include an audiovisual medium and an instrument developed especially for this study to achieve a greater impact on its target audience. It is necessary to continue with this line of research in other populations and consider perceptions and behaviors as variables of interest; these results will result in a better understanding of environmental care and, therefore, in the development of our city and the country.

Limitations:

A limitation of the study was the little information available in dentistry about caring for the environment, which did not allow many comparisons to be made with other studies. Likewise, it was difficult to access to conduct interviews with academics and authorities representing institutions related to this topic in the environment, which caused the extension in the execution of the educational video.

## CONCLUSIONS

In the pre-application of the educational program, women and fifth semester students presented better scores in attitudes and knowledge, unlike in the post-application of the program where no influence of gender was found in any of the scores.

The present investigation concludes that the Online Educational Program: Care of the Environment in the Dental field, was effective in improving attitudes and knowledge according to the comparisons made between pre and post application.
